# Supplementary material for: A Likelihood Approach for Real-Time Calibration of Stochastic Compartmental Epidemic Models
Source: PLoS Comput Biol. 2017 Jan 17;13(1):e1005257. doi: 10.1371/journal.pcbi.1005257 (PMC5240920; doi:10.1371/journal.pcbi.1005257)
Supplement: S1 File — (TAR.GZ) [file pcbi.1005257.s014.tar.gz › HSPH_Online-SI-Revision/PF08/n1-mild/n1-mild_PF_table-4.pdf]

1	R0	{1.27677, 7.89375}	{1.80874, 14.8229}	{{1.25604, 7.74295}, {2.41537, 20.}}	{42.9345, 90.901}	inf	17.	101.807	{31.0833, 209.237}	499.688	0.672103	newly	24.	47.93	{16., 90.}	114.958	0.294557	Reff	1.16697	1.15269	{0.682934, 1.5881}	23.519	ar	400.	811.9	{566., 1021.}	102.975
2	R0	{1.33231, 10.5259}	{1.64297, 12.8584}	{{1.11461, 5.72868}, {2.42205, 19.0443}}	{32.6614, 42.5372}	inf	39.	47.1867	{2.69113, 103.678}	85.5181	0.471628	newly	28.	21.38	{2., 46.}	56.6429	0.43651	Reff	0.927288	0.778373	{0.395005, 1.0551}	28.4236	ar	467.	581.29	{259., 878.}	47.9251
3	R0	{1.2273, 6.76944}	{1.81865, 13.2115}	{{1.10693, 6.31117}, {2.73737, 18.2127}}	{52.2897, 100.889}	inf	19.	79.0251	{5.60636, 187.755}	342.348	0.610515	newly	22.	45.1	{4., 98.}	146.091	0.492402	Reff	1.11071	1.36451	{0.896103, 2.0572}	31.9257	ar	314.	615.56	{172., 903.}	115.389
4	R0	{1.41215, 17.5742}	{1.72907, 14.9713}	{{1.23554, 7.41331}, {2.39415, 20.}}	{28.4952, 23.1578}	inf	31.	88.6566	{30.7498, 174.762}	192.246	0.403366	newly	15.	42.99	{16., 73.}	191.133	0.397641	Reff	1.27376	1.1285	{0.738646, 1.65134}	25.5912	ar	465.	764.67	{524., 994.}	65.671
5	R0	{1.42222, 6.15886}	{1.6301, 13.2258}	{{1.12882, 6.18069}, {2.22832, 19.0269}}	{25.353, 119.278}	inf	27.	70.2547	{4.38824, 155.325}	196.675	0.518297	newly	31.	34.69	{3., 68.}	65.9032	0.386182	Reff	1.21173	1.02442	{0.654667, 1.4114}	26.0765	ar	490.	574.16	{202., 843.}	43.0735
6	R0	{1.24363, 9.95596}	{1.80221, 13.2186}	{{1.11228, 6.19362}, {2.73737, 18.4249}}	{48.7862, 48.1599}	inf	12.	75.9671	{10.6529, 159.843}	545.162	0.680981	newly	7.	44.15	{5., 94.}	543.286	0.693918	Reff	1.21503	1.33559	{0.862846, 1.83165}	26.4022	ar	411.	619.2	{197., 903.}	70.8078
7	R0	{1.36478, 5.96056}	{1.82342, 13.0037}	{{1.07469, 6.0993}, {2.77502, 18.4249}}	{41.3701, 121.757}	inf	11.	87.2017	{7.99636, 202.343}	707.711	0.759305	newly	13.	44.8	{4., 97.}	270.923	0.556184	Reff	1.2092	1.22432	{0.723204, 1.79608}	27.991	ar	484.	639.14	{214., 917.}	56.3678
8	R0	{1.36335, 17.6429}	{1.52032, 13.5119}	{{1.22568, 6.3986}, {1.9308, 20.}}	{16.8728, 28.7921}	inf	30.	55.7063	{11.4513, 99.2584}	110.691	0.344287	newly	14.	25.85	{8., 45.}	100.5	0.295452	Reff	1.27473	0.925397	{0.648052, 1.22346}	28.8396	ar	484.	691.46	{506., 904.}	43.8471
9	R0	{1.26575, 5.28707}	{1.61862, 13.9291}	{{1.17174, 7.03175}, {2.09752, 20.}}	{30.5005, 165.919}	inf	13.	60.7917	{10.7334, 127.471}	378.144	0.600255	newly	20.	26.98	{8., 54.}	67.2	0.268017	Reff	1.05943	0.846948	{0.559882, 1.13277}	25.0921	ar	472.	730.31	{480., 961.}	55.4979
10	R0	{1.42942, 9.49905}	{1.64083, 14.381}	{{1.20234, 8.88983}, {2.09665, 20.}}	{21.7968, 58.2134}	inf	45.	79.1545	{27.6135, 147.798}	94.6669	0.267839	newly	34.	36.51	{15., 65.}	44.0882	0.196136	Reff	1.07635	0.913686	{0.614045, 1.22023}	22.4586	ar	468.	755.37	{533., 976.}	62.0833
11	R0	{1.46827, 15.0983}	{1.56266, 13.318}	{{1.20167, 6.50141}, {1.94423, 20.}}	{17.476, 29.7981}	inf	28.	56.4212	{2.81846, 122.656}	131.62	0.4302	newly	14.	26.06	{5., 50.}	109.429	0.338174	Reff	1.36843	0.83681	{0.537054, 1.08699}	38.8488	ar	495.	698.98	{487., 900.}	43.4505
12	R0	{1.25751, 13.279}	{1.53178, 13.4598}	{{1.19541, 6.69555}, {2.03225, 20.}}	{23.786, 33.002}	inf	18.	57.6902	{11.6338, 107.658}	236.273	0.493298	newly	8.	27.39	{9., 53.}	250.625	0.496276	Reff	1.17829	0.904226	{0.578823, 1.16801}	24.7943	ar	366.	680.85	{456., 942.}	86.2978
13	R0	{1.3425, 9.16361}	{1.64509, 12.8371}	{{1.11164, 6.05643}, {2.42205, 18.9813}}	{32.5243, 54.9645}	inf	32.	49.2057	{2.81822, 103.84}	106.527	0.453928	newly	28.	22.58	{3., 49.}	52.0714	0.374964	Reff	0.965258	0.807705	{0.428419, 1.08442}	29.0672	ar	442.	576.47	{229., 870.}	54.9027
14	R0	{1.18936, 6.20583}	{1.68707, 13.4799}	{{1.11228, 5.72868}, {2.45983, 19.0269}}	{44.6971, 124.124}	inf	26.	78.3715	{2.39468, 181.44}	239.863	0.596169	newly	32.	38.17	{3., 81.}	76.6563	0.457508	Reff	1.01333	1.03717	{0.731017, 1.4299}	22.8601	ar	382.	584.5	{201., 871.}	73.0157
15	R0	{1.27137, 9.78868}	{1.64687, 13.2675}	{{1.1298, 6.05643}, {2.24606, 19.255}}	{34.394, 51.3359}	inf	27.	66.2674	{4.92125, 135.575}	184.125	0.494168	newly	24.	32.95	{3., 65.}	86.875	0.415894	Reff	1.10101	1.06326	{0.713138, 1.53048}	24.4917	ar	496.	573.06	{184., 841.}	42.7903
16	R0	{1.23102, 13.6452}	{1.6943, 14.8456}	{{1.23061, 8.08683}, {2.39424, 20.}}	{38.3167, 28.0028}	inf	33.	99.5257	{31.969, 220.247}	207.014	0.398147	newly	16.	45.03	{16., 91.}	185.313	0.389545	Reff	1.07222	1.07621	{0.747487, 1.40556}	18.8349	ar	445.	762.39	{536., 1026.}	72.2629
17	R0	{1.27213, 13.7452}	{1.62256, 13.0474}	{{1.11365, 6.05643}, {2.22212, 18.9248}}	{32.9326, 29.3586}	inf	22.	63.159	{2.14702, 146.457}	224.549	0.567558	newly	11.	34.04	{1., 69.}	241.091	0.582032	Reff	1.19962	1.12523	{0.76063, 1.59714}	24.8336	ar	367.	575.97	{245., 850.}	71.9319
18	R0	{1.25318, 11.2151}	{1.57136, 14.7355}	{{1.17246, 7.88988}, {2.06271, 20.}}	{28.0732, 43.3426}	inf	26.	68.3016	{14.0539, 129.789}	178.65	0.404968	newly	13.	27.99	{9., 52.}	128.077	0.330079	Reff	1.10029	0.831457	{0.532073, 1.04633}	25.1512	ar	416.	710.27	{457., 924.}	71.5361
19	R0	{1.46006, 8.11496}	{1.7986, 13.1875}	{{1.13239, 6.0993}, {2.52651, 18.9813}}	{32.9998, 72.5084}	inf	42.	77.9796	{5.40768, 175.555}	131.818	0.488451	newly	40.	40.03	{7., 83.}	60.225	0.418365	Reff	1.24251	1.1849	{0.738134, 1.77136}	31.7755	ar	496.	636.59	{244., 889.}	51.4173
20	R0	{1.14273, 6.71261}	{1.77848, 14.8932}	{{1.25622, 7.77081}, {2.77027, 20.}}	{55.6339, 124.112}	inf	15.	97.7882	{30.7044, 216.294}	552.132	0.712896	newly	12.	45.43	{17., 83.}	279.25	0.509938	Reff	1.02046	1.07815	{0.696884, 1.54699}	26.01	ar	362.	780.76	{507., 1031.}	115.68
21	R0	{1.42432, 8.23905}	{1.60371, 12.4196}	{{1.11523, 5.32461}, {2.40085, 18.3794}}	{25.8062, 64.8347}	inf	17.	58.9072	{3.49863, 124.099}	279.031	0.564368	newly	14.	28.43	{2., 59.}	142.5	0.438244	Reff	1.15512	0.877997	{0.536784, 1.17306}	29.4074	ar	405.	563.61	{242., 866.}	55.9531
22	R0	{1.29386, 17.863}	{1.71399, 14.5112}	{{1.2189, 8.96715}, {2.38141, 20.}}	{34.4338, 22.9438}	inf	27.	89.0613	{28.2408, 183.229}	232.613	0.425329	newly	8.	45.39	{14., 86.}	468.125	0.658567	Reff	1.20588	1.20605	{0.799343, 1.66998}	18.3938	ar	488.	767.62	{527., 987.}	59.4303
23	R0	{1.39274, 13.0167}	{1.64335, 14.655}	{{1.20851, 7.61116}, {2.17366, 20.}}	{24.898, 31.6412}	inf	34.	72.205	{23.1354, 128.568}	128.442	0.326832	newly	12.	31.4	{11., 49.}	170.333	0.391564	Reff	1.12533	0.880476	{0.551005, 1.15582}	24.517	ar	475.	744.44	{499., 1001.}	57.8232
24	R0	{1.32178, 15.8991}	{1.6179, 14.0453}	{{1.20789, 7.36321}, {2.22754, 20.}}	{26.0795, 27.0978}	inf	43.	79.081	{26.951, 144.569}	104.859	0.290768	newly	19.	37.09	{14., 63.}	105.632	0.280184	Reff	1.16978	1.00506	{0.667952, 1.28726}	20.9225	ar	471.	731.24	{517., 966.}	56.1996
25	R0	{1.37568, 17.8427}	{1.62008, 13.7101}	{{1.20669, 5.28305}, {2.21025, 20.}}	{24.2923, 28.4306}	inf	18.	64.2219	{8.13977, 146.307}	277.693	0.521692	newly	6.	26.84	{7., 56.}	355.667	0.582334	Reff	1.20647	0.830602	{0.473436, 1.1306}	31.8308	ar	398.	725.38	{484., 955.}	82.3568
26	R0	{1.20993, 6.4656}	{1.64179, 14.9309}	{{1.2348, 8.48113}, {2.0868, 20.}}	{36.1492, 131.859}	inf	13.	81.0333	{27.9116, 140.557}	523.333	0.714837	newly	16.	36.99	{15., 66.}	135.438	0.323646	Reff	1.03933	1.05947	{0.722298, 1.36381}	21.4089	ar	345.	756.88	{510., 943.}	119.386
27	R0	{1.20548, 13.8003}	{1.5275, 14.0719}	{{1.18097, 6.6536}, {1.90314, 20.}}	{27.7562, 32.1134}	inf	42.	61.7788	{4.94163, 126.518}	82.6488	0.354204	newly	24.	27.73	{6., 51.}	59.2917	0.298284	Reff	1.0548	0.877149	{0.619918, 1.12365}	20.4112	ar	350.	698.56	{482., 888.}	99.6286
28	R0	{1.3176, 14.6594}	{1.69639, 14.483}	{{1.20092, 8.17676}, {2.36708, 20.}}	{32.7977, 23.6533}	inf	14.	80.93	{24.5664, 173.452}	478.071	0.645151	newly	4.	41.25	{13., 83.}	931.25	0.91419	Reff	1.27412	1.22153	{0.855847, 1.70484}	19.9736	ar	362.	733.96	{450., 984.}	104.619
29	R0	{1.28202, 6.23047}	{1.70563, 14.6927}	{{1.20628, 7.72427}, {2.4565, 20.}}	{35.3805, 137.517}	inf	36.	90.9241	{31.8534, 190.241}	160.523	0.34802	newly	35.	41.32	{14., 76.}	53.6	0.213951	Reff	1.10382	0.995519	{0.660187, 1.27763}	21.1734	ar	391.	758.55	{491., 1024.}	94.3095
30	R0	{1.28177, 8.83252}	{1.73206, 13.3358}	{{1.11228, 5.62019}, {2.59907, 19.0269}}	{40.9232, 67.0725}	inf	30.	77.4154	{3.91537, 175.555}	200.694	0.550478	newly	21.	38.11	{4., 83.}	122.143	0.469732	Reff	1.12539	1.15471	{0.748712, 1.65342}	26.9492	ar	427.	608.8	{244., 902.}	62.8712
31	R0	{1.35886, 5.97708}	{1.64114, 13.2401}	{{1.1298, 6.62607}, {2.24606, 19.0443}}	{28.838, 126.093}	inf	23.	64.7004	{2.99155, 143.199}	218.183	0.535062	newly	44.	31.71	{3., 63.}	54.1591	0.465169	Reff	1.14008	1.02664	{0.698749, 1.5291}	26.7874	ar	491.	583.55	{225., 842.}	44.6578
32	R0	{1.27601, 17.9804}	{1.66381, 13.7898}	{{1.2031, 7.0034}, {2.27853, 20.}}	{32.6112, 27.9602}	inf	25.	81.7713	{11.6895, 167.227}	242.	0.477438	newly	8.	37.4	{12., 67.}	368.75	0.591104	Reff	1.18924	0.979319	{0.605143, 1.2584}	23.4266	ar	445.	736.5	{477., 975.}	66.0674
33	R0	{1.3368, 11.3235}	{1.72196, 14.7073}	{{1.23894, 7.3214}, {2.60189, 20.}}	{32.0668, 44.1581}	inf	40.	88.5042	{28.4874, 178.247}	133.111	0.313524	newly	21.	42.56	{17., 71.}	111.524	0.284542	Reff	1.1697	1.0961	{0.684496, 1.6011}	23.2441	ar	417.	768.67	{512., 1023.}	84.5012
34	R0	{1.21021, 11.3635}	{1.71987, 14.6818}	{{1.24882, 8.18676}, {2.38729, 20.}}	{42.4908, 41.5372}	inf	20.	84.4922	{26.8682, 171.443}	323.631	0.53066	newly	14.	38.62	{16., 70.}	177.429	0.38154	Reff	1.06862	1.02376	{0.583668, 1.36014}	23.0358	ar	327.	769.55	{492., 1014.}	135.41
35	R0	{1.53382, 10.8866}	{1.76976, 14.978}	{{1.22615, 8.99029}, {2.62238, 20.}}	{26.6828, 45.8479}	inf	30.	98.263	{30.949, 186.594}	230.66	0.431804	newly	22.	46.08	{17., 88.}	120.182	0.306028	Reff	1.3789	1.12568	{0.735922, 1.4929}	26.0851	ar	476.	786.91	{511., 1020.}	66.187
36	R0	{1.20423, 6.83844}	{1.70168, 13.404}	{{1.12882, 6.18069}, {2.24606, 18.834}}	{44.1292, 102.78}	inf	44.	74.751	{5.7877, 167.7}	118.277	0.479689	newly	49.	38.47	{5., 83.}	53.4898	0.435415	Reff	1.05852	1.14551	{0.75127, 1.59714}	26.9868	ar	416.	613.51	{256., 862.}	64.3389
37	R0	{1.43629, 10.1548}	{1.61874, 14.7622}	{{1.21462, 7.41813}, {2.15818, 20.}}	{21.9119, 58.9784}	inf	15.	78.4312	{26.356, 134.695}	424.816	0.631486	newly	13.	36.55	{16., 61.}	181.769	0.392012	Reff	1.27255	1.06206	{0.742558, 1.35314}	22.6485	ar	367.	731.68	{491., 984.}	100.022
38	R0	{1.37504, 8.93428}	{1.79601, 13.5455}	{{1.15921, 6.0993}, {2.52651, 19.1171}}	{37.0801, 63.2564}	inf	32.	77.9466	{3.91537, 175.555}	182.747	0.537271	newly	24.	38.59	{4., 80.}	100.542	0.457954	Reff	1.17703	1.18052	{0.738134, 1.75555}	31.065	ar	483.	634.6	{244., 889.}	53.9089
39	R0	{1.51637, 9.36742}	{1.65248, 13.1158}	{{1.12882, 6.18069}, {2.29738, 19.0355}}	{24.6538, 54.3474}	inf	43.	64.798	{5.34355, 141.756}	105.07	0.449878	newly	31.	31.19	{4., 63.}	63.3226	0.380926	Reff	1.204	0.955685	{0.515248, 1.3121}	30.587	ar	441.	585.93	{238., 857.}	54.9683
40	R0	{1.23658, 9.82869}	{1.72692, 13.0656}	{{1.05756, 6.31117}, {2.59602, 18.9813}}	{44.7238, 47.989}	inf	29.	72.6435	{3.85453, 166.495}	192.945	0.532499	newly	15.	36.82	{4., 75.}	178.	0.495454	Reff	1.09561	1.22759	{0.781251, 1.78306}	30.9586	ar	414.	595.2	{182., 902.}	67.8454
41	R0	{1.24247, 5.4242}	{1.7644, 13.2111}	{{1.07662, 6.8071}, {2.46976, 17.8178}}	{46.5613, 145.795}	inf	23.	81.8828	{4.99197, 202.343}	288.654	0.576307	newly	36.	43.96	{3., 97.}	82.3889	0.45569	Reff	1.1381	1.2207	{0.798891, 1.74375}	26.6407	ar	345.	619.38	{213., 893.}	98.8522
42	R0	{1.28208, 4.40223}	{1.81686, 13.4381}	{{1.14345, 6.0993}, {2.59907, 19.0269}}	{46.0075, 206.405}	inf	20.	71.8802	{3.85453, 165.044}	290.73	0.594272	newly	30.	37.34	{4., 75.}	78.	0.448407	Reff	1.1949	1.32833	{0.789151, 1.96354}	32.8676	ar	339.	636.41	{240., 906.}	103.466
43	R0	{1.56773, 6.50474}	{1.81103, 13.0482}	{{1.21278, 6.74711}, {2.47422, 18.4377}}	{28.7651, 105.938}	inf	33.	73.8668	{3.61356, 163.043}	166.175	0.518345	newly	44.	42.95	{2., 90.}	65.2955	0.481422	Reff	1.34982	1.37256	{0.843355, 1.93866}	25.3574	ar	498.	638.68	{327., 893.}	49.0763
44	R0	{1.10489, 6.01941}	{1.63618, 13.8531}	{{1.20808, 7.14345}, {2.14005, 20.}}	{48.085, 133.783}	inf	12.	82.3686	{24.9375, 170.294}	594.296	0.735518	newly	14.	36.66	{14., 64.}	168.714	0.379951	Reff	0.942471	0.926296	{0.606106, 1.19546}	20.053	ar	346.	734.63	{506., 967.}	112.627
45	R0	{1.25888, 14.1926}	{1.60464, 14.5331}	{{1.188, 8.31785}, {2.09775, 20.}}	{29.4412, 25.6327}	inf	50.	63.3131	{23.5245, 106.744}	56.9949	0.21635	newly	23.	28.66	{13., 48.}	49.913	0.189524	Reff	0.981926	0.878118	{0.503197, 1.1831}	22.5392	ar	471.	727.07	{493., 944.}	55.1953
46	R0	{1.45292, 16.616}	{1.64341, 13.9693}	{{1.07874, 6.16288}, {2.25463, 19.0443}}	{26.5338, 24.0042}	inf	34.	68.1222	{2.14702, 147.091}	153.093	0.546979	newly	17.	33.87	{1., 74.}	144.294	0.513146	Reff	1.34105	1.12609	{0.762641, 1.56866}	25.8065	ar	494.	576.26	{244., 851.}	44.3077
47	R0	{1.22528, 16.6033}	{1.63092, 14.7588}	{{1.21505, 8.89073}, {2.34066, 20.}}	{33.9254, 22.1673}	inf	14.	77.9662	{24.5448, 181.161}	456.902	0.635022	newly	6.	37.15	{13., 73.}	519.167	0.700518	Reff	1.17014	1.14919	{0.765198, 1.42291}	18.7347	ar	368.	724.13	{467., 966.}	96.8342
48	R0	{1.1669, 6.92715}	{1.56409, 14.1462}	{{1.19175, 7.04311}, {2.15745, 20.}}	{34.3519, 108.639}	inf	17.	58.6252	{12.4803, 110.747}	256.586	0.500557	newly	17.	26.49	{9., 46.}	78.4118	0.263449	Reff	0.977862	0.888189	{0.535447, 1.15436}	20.3452	ar	416.	707.25	{501., 963.}	70.4495
49	R0	{1.32749, 16.0887}	{1.63217, 14.3051}	{{1.20512, 7.35444}, {2.39951, 20.}}	{27.287, 24.4393}	inf	24.	78.8554	{25.2827, 176.076}	234.391	0.431459	newly	8.	37.14	{13., 75.}	364.5	0.584359	Reff	1.24917	1.06163	{0.704031, 1.32315}	21.1448	ar	353.	713.56	{465., 996.}	102.142
50	R0	{1.29636, 15.2609}	{1.61213, 14.0293}	{{1.2168, 7.60534}, {2.10416, 20.}}	{27.0414, 28.0643}	inf	50.	70.2264	{15.3071, 132.334}	76.9418	0.308928	newly	20.	31.59	{11., 49.}	79.95	0.263916	Reff	1.03838	0.877982	{0.555551, 1.13061}	22.2141	ar	470.	735.91	{495., 956.}	58.1936
